# Atomic diffraction by nanoholes in hexagonal boron nitride†

**DOI:** 10.1039/d4na00322e

**Published:** 2024-09-02

**Authors:** Eivind Kristen Osestad, Ekaterina Zossimova, Michael Walter, Bodil Holst, Johannes Fiedler

**Affiliations:** a Department of Physics and Technology, University of Bergen Allégaten 55 5007 Bergen Norway johannes.fiedler@uib.no; b Lace Lithography AS Allégaten 55 5007 Bergen Norway; c Department of Physics and Astronomy, Living Systems Institute, University of Exeter EX4 4QD Exeter UK; d Freiburg Center for Interactive Materials and Bioinspired Technologies (FIT), University of Freiburg D-79110 Freiburg Germany; e Institute of Physics, University of Freiburg Hermann-Herder-Str. 3 79104 Freiburg Germany

## Abstract

Fabricating patterned nanostructures with matter waves can help to realise new nanophotonic devices. However, due to dispersion effects, designing patterns with nanoscale features is challenging. Here, we consider the propagation of a helium matter wave through different holes in hexagonal boron nitride (h-BN) as a case study for the weakest dispersion interaction and the matter wave's diffraction as it passes through the holes. We use a quantum-mechanical model to calculate the polarisability of edge atoms around the holes, where we observe polarisation ripples of enhanced and reduced polarisabilities around the holes. We use these values to calculate van der Waals dispersion coefficients for the scattered helium atoms. We find that the resulting diffraction patterns are affected by the shape and size of the holes, where the smallest holes have a radius of just 6 Å. These results can be used to predict the resolution limits of nano-hole patterns on nanophotonic materials.

## Abstract 2

This theoretical study explores the capability to create nanostructures using matter waves, focusing on helium diffraction through h-BN holes. A quantum-mechanical model reveals how edge atom polarizability and hole size affect diffraction patterns.

## Graphical abstract

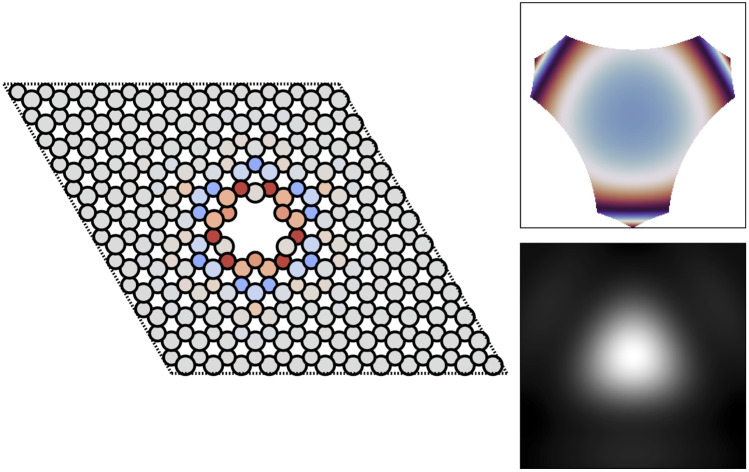

## 1 Introduction

Atomic interferometry is useful for precise measurement that can be applied in fundamental physics tests^1^ and accurate inertial sensing.^2^ It is common to use material gratings in such experiments in order to make the atom interfere.^3^ For very small holes or slow atoms, dispersion forces between the interfering atoms and the mask begin to have a significant effect. Examples of such forces include the van der Waals force between neutral particles and the Casimir–Polder force between neutral particles and dielectric materials.^4,5^ These forces arise from ground state fluctuations of the electromagnetic fields and reduce the effective size of the holes, as shown in Fig. 4. Furthermore, they produce a phase shift that affects the atomic waves passing through the holes.^6^ This limits the size of the holes in a given diffraction mask.

Since the dispersion forces depend on the thickness of the mask, 2D monolayer materials, such as hexagonal boron nitride (h-BN)^7^ and graphene,^8,9^ represent the theoretical lower limit to these interactions. Graphene has already been used as a beam splitter for matter waves in experiments by creating lines and holes in it.^8^ In addition, by firing high-speed hydrogen and helium atoms at the membrane (at a speed in excess of 27 000 ms^−1^), the matter wave should semi-coherently diffract through even the hexagonal grid in 2D materials.^10^

Many advanced technologies exploit quantum effects at the nanoscale, requiring highly controlled and precise fabrication techniques. Examples include quantum electronic devices, such as resonant tunnelling diodes, single-electron Coulomb blockade transistors,^11,12^ and quantum dot transistors.^13^ However, fabrication techniques often limit the realisation of new devices. For example, ferromagnetic semiconductors could be used in the next generation of energy-efficient computers and electronic devices, which rely on the quantum control of spin states instead of charge carriers.^14^ However, the fabrication of ferromagnetic semiconductors is challenging and requires new methods to pattern magnetic materials as sub-nanometre dots.

Currently, it is only possible to pattern arbitrary structures with resolution and pitch on the “few-nanometre” scale using electron or ion beam lithography or a scanning probe tip.^15^ These techniques all write patterns in a series, one pixel at a time, which makes them unsuitable for large-scale industrial applications as it is too time-consuming to pattern large areas. Thus, the lithography industry is dominated by photolithography due to its much higher speed despite its lower resolution. The current state of the art is extreme-ultra-violet (EUV) lithography. This uses light with a wavelength of 13.5 nm, corresponding to an Abbe resolution limit of 6.75 nm, assuming the maximum value of the numerical aperture (NA = 1). Beyond the Abbe resolution limit, electron blurring of the pattern from secondary effects in the resist material limits the ultimate resolution to approximately 6 nm for any photon-based lithography.^16^

Binary holography methods using metastable atoms have been proposed as a solution to the challenge of speeding up and improving the resolution of this process.^17–19^ The early proposals did not account for dispersion forces between atoms and the mask.^20,21^ This is a problem, as when using binary holography, very small holes are needed to create patterns of high resolution,^17,18^ and thus, these forces must be accounted for. A structure of holes in a membrane, a so-called “atom sieve”, has been used to focus neutral helium atoms, analogous to the “photon sieve” solution^22^ for the purpose of neutral helium atom microscopy.^23^

In the case of h-BN considered here, additional electrostatic forces may appear near the hole as the single charges do not compensate for each other. These forces are attractive and have a smaller but similar effect on surrounding atoms compared to the dispersion forces.^24^

In this paper, we present a model describing the diffraction of a neutral, ground-state helium matter wave through a monolayer. More specifically, we consider helium atoms passing holes in h-BN. We chose these materials as they are both available for experiments, and they both have weak dispersion interactions due to helium being a small, inert atom and h-BN being an insulator. Thus, the dispersion interaction will be weak. By removing atoms from the lattice structure of the h-BN monolayer, we create four stable holes that vary in size and shape from about 5–15 Å across. The experimental realisation of atomically precise holes has been demonstrated using electron beams,^25^ proton beams^26^ and ions.^27^ By modelling the atoms quantum mechanically, we find the forces acting on the atoms passing through the hole and the effective reduction in the radii of the holes. Then, we use the hole reduction and the forces to calculate the diffraction patterns macroscopically.

First, in Section 2, we use electronic structure theory and dispersion force theory to model defects in h-BN. We find several stable structures corresponding to charge-compensated holes in h-BN. We determine the polarisability and van der Waals coefficients of edge atoms around the defects, which allows us to calculate the corresponding forces around the hole. Then, in Section 3.1, we estimate the hole reduction by simulating a helium atom colliding with boron and nitrogen atoms. Having found both the forces and the hole reduction, we switch to a macroscopic diffraction picture in Section 3.3, due to the difficulty of numerically modelling the atomic wavefunction. We calculate the resulting shape of the hole and how the forces inside and outside the hole change the phase of the matter wave passing through it. Using this information, we find a transmission function, telling us where the atom can pass through the hole and the phase change at that point. This can then be used to find the far-field diffraction patterns of the holes.

## 2 Modelling

### 2.1 Screened atomic polarisabilities

We obtain the screened atomic polarisabilities from the electronic structure theory. In the first step, the structures of pristine h-BN and several defects are constructed with the help of the atomic simulation environment.^28^ h-BN has a hexagonal lattice structure, similar to graphene, with a lattice constant *a*_l_ = 0.2504 nm,^29,30^ leading to a bond-length between boron and nitrogen of *a* = 0.1446 nm. We consider only neutral holes where the number of removed B and N atoms is equal in order to avoid the need for compensation charges in periodic calculations. The resulting structure is then relaxed until the maximal force on each atom is below 0.05 eV Å^−1^. The electronic structure is determined within DFT as implemented in the open-source GPAW code.^31,32^ The exchange–correlation energy is described by the PBE functional.^33^ Kohn–Sham wave functions and electron density are represented in Blöchl's projector augmented wave method^34^ and the smooth wave functions are represented on real space grids with a grid spacing of 0.2 Å.

We follow the Tkatchenko–Scheffler approach^35,36^ to correct the atomic polarisabilities for effects through the constraints of their interaction within the material. Free atomic polarisabilities, *α*^free^_*i*_, are taken from the Chu and Dalgarno dataset^37^ and are isotropic. The polarisability of the bounded atoms, *α*^hirsh^_*i*_, are known to scale approximately linearly^38^ with the ratios of bonded-atom volumes to free atom volumes1
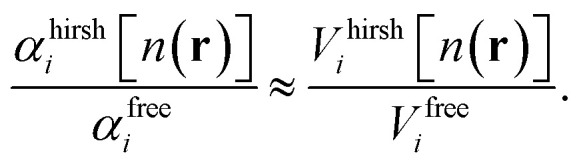
The volume ratios, *V*^hirsh^_*i*_/*V*^free^_*i*_, are calculated from the electronic density of the h-BN monolayer, [*n*(**r**)], using the Hirshfeld charge partitioning scheme.^39^

We subsequently apply a correction for screening between neighbouring atoms using the range-separated self-consistent screening method available in the libMBD code.^40,41^ Since the h-BN supercell is periodic, a cut-off radius determines neighbouring atoms, and calculations are truncated according to the Ewald summation method.^42,43^ The screened atomic polarisabilities are obtained by solving the self-consistent screening equation from classical electrodynamics2
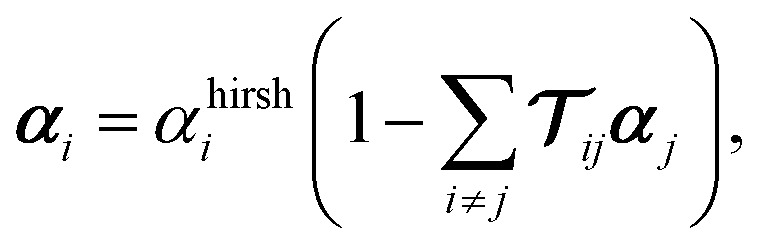
where ***α***_*i*_ denote the screened atomic polarisability tensors and *α*^hirsh^_*i*_ are the Hirshfeld-partitioned atomic polarisabilities given by eqn (1). Here, 
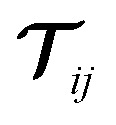
 denotes the dipole–dipole interaction tensor, which depends on the relative displacement of the atoms in the h-BN monolayer. The implementation in the libMBD code attenuates the short-range interactions between atoms to avoid unphysical values at short interatomic separations.

### 2.2 Screened van der Waals coefficients

Calculating the van der Waals dispersion coefficients requires a model for the dynamic polarisability of atoms. We can introduce frequency dependence through the single-pole approximation3
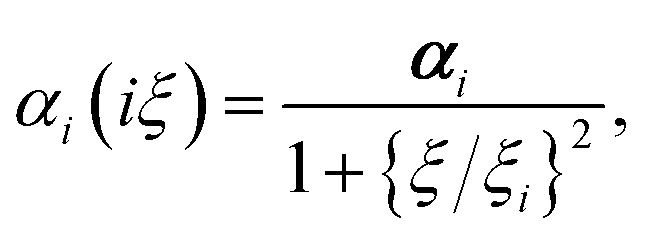
where *α*_*i*_ = Tr[***α***_*i*_]/3 denotes the scalar, screened polarisability of an atom in the h-BN membrane, evaluated using eqn (2). *ξ*_*i*_ is the corresponding characteristic resonance frequency, approximated by^35^4
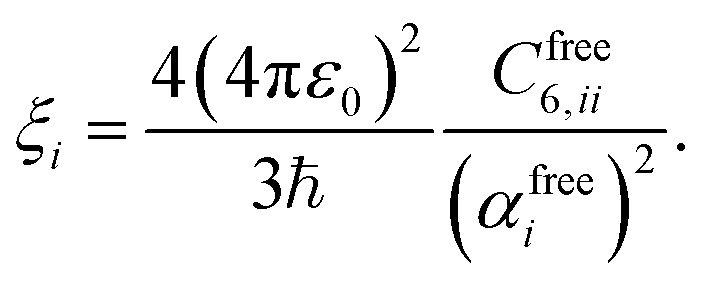
using the free atom values for the polarisabilities and van der Waals *C*_6_ coefficients.^37^ This represents a suitable approximation for *ξ*_*i*_ because the scaling factors due to Hirshfeld partitioning approximately cancel each other in this ratio.

The screened van der Waals coefficients can then be obtained using the Casimir–Polder integral,5
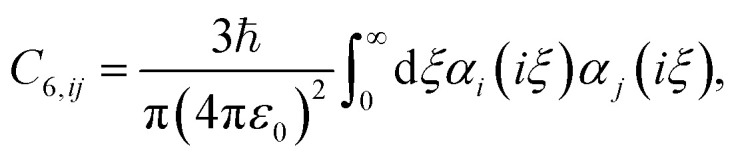
which can be solved by substituting eqn (3) into (5). This leads to the London formula,^35,44^6
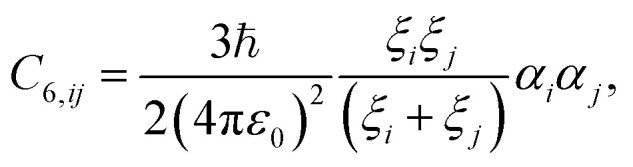
in terms of the screened atomic polarisabilities *α*_*i*_ = Tr[***α***_*i*_]/3 and characteristic frequencies *ξ*_*i*_.

Here, we are interested in the dispersion interactions between free helium particles and the set of {*j*} atoms in the h-BN membrane. Therefore, we can set *ξ*_*i*_ = *ξ*^He^ and *α*_*i*_ = *α*^He^ in eqn (6), reducing the dimensionality of the problem. This gives7
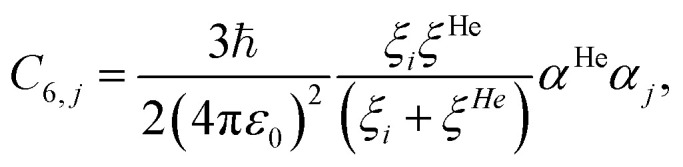
where the values for the free helium atom are given in Table 1. The values of *α*_*j*_ depend on the atomic species and the displacement of the atom relative to the defect in the monolayer.

**Table 1:** Polarisability and (homonuclear) *C*_6_ coefficients (in atomic units) of boron and nitrogen atoms in the infinite h-BN monolayer and the free helium atom. 1 Bohr^3^ = 1.48 × 10^−31^ m^3^, 1 Ha Bohr^6^ = 9.57 × 10^−80^ J m^6^, 1 Ha = 4.36 × 10^−18^ J

Atom	*α*/(4π*ε*_0_) [Bohr^3^]	*C*_6_ [Ha Bohr^6^]	ℏ*ξ* [Ha]
B	18.09	75.23	0.30
N	3.70	14.62	0.59
He	1.38	1.42	0.99

### 2.3 Polarisability ripples

**Fig. 1:**
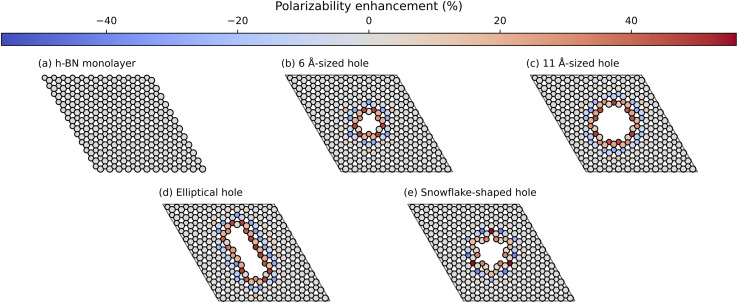
Polarisability ripples around different shaped holes in the h-BN monolayer: (a) monolayer, (b) 6 Å-sized hole, (c) 11 Å-sized hole, (d) elliptic hole, and (e) snowflake-shaped hole. The colour bar represents the percentage increase (red) or decrease (blue) of the atomic polarisabilities compared to equivalent atoms in the infinite h-BN monolayer. Geometries visualised with the Atomic Simulation Environment^28^ and overlaid with polarisability data using Matplotlib.^45^

We solve eqn (2) for the 5 different supercell geometries shown in Fig. 1. The structures are charge neutral since we remove equal numbers of boron and nitrogen atoms to create the holes. The infinite h-BN monolayer is used as a reference point to calculate the change in atomic polarisabilities for the other structures with differently shaped holes. The baseline values for the infinite h-BN supercell are summarised in Table 1. In the case of supercells with holes, we observe a “polarisability ripple” around the hole's circumference. The atoms immediately surrounding the hole have an enhanced polarisability compared to equivalent atoms in the infinite h-BN monolayer. The enhancement is about twice as strong for nitrogen atoms (≈40%) compared to boron atoms (≈20%). The second ring of atoms surrounding the hole shows a decrease in atomic polarisability, although this is a less pronounced change than for the first ring of atoms. This ripple effect propagates outwards from the hole, and the oscillations rapidly decay until they return to the h-BN baseline values for the infinite monolayer. This effect looks like a “polarisability ripple” in Fig. 1(b)–(e).

### 2.4 Dispersion interactions by h-BN monolayers

**Fig. 2:**
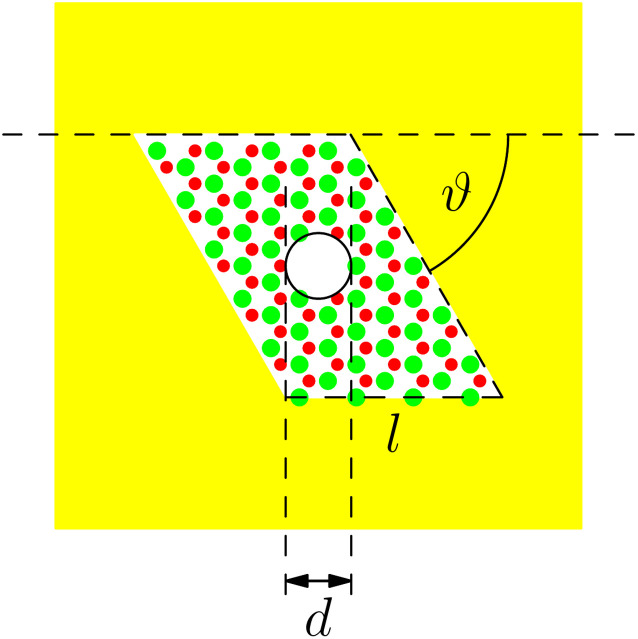
Sketch of a spherical hole with diametre *d* in an infinite monolayer (yellow area). A small rhombus with side length *l* and angle *ϑ* will be treated atomically. The remaining yellow area is a solid object. We always model a large enough area atomically such that we can neglect the solid part.

We now determine the interaction potentials experienced by a helium atom approaching h-BN monolayers. We consider an atomically thin monolayer as depicted in Fig. 2. A small spherical hole with a diameter *d* is treated as a vacancy of several atoms. Thus, the interaction is considered on an atomic level in a rhombus with the side length *l* surrounding the hole. The remaining part of the monolayer membrane is treated as a continuum without significant interaction. We particularly consider hexagonal boron nitride (h-BN) and neutral helium atoms. However, the derived models for the interactions and their effective treatments for matter-wave interference can also be adapted easily to other materials.

The interaction between a neutral particle characterised through its polarisability tensor **α** and a dielectric object is, in general, given by the Casimir–Polder potential^4,20^8
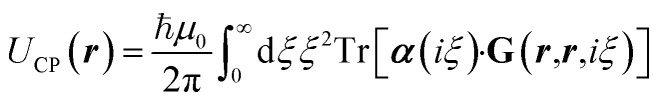
with the reduced Planck constant ℏ, the vacuum permeability *μ*_0_ and the scattering Green function **G** that contains the properties of the dielectric object. This equation can be understood as an exchange of virtual photons with frequency *iξ*, which are induced at the particle's position ***r*** and back-scattered from the surrounding dielectric objects as described by **G**(***r***,***r***,*iξ*). The scattered virtual photons polarise the particle. The sum (respectively the integral) over all possible photon exchanges yields the Casimir–Polder interaction.

Here, for a monolayer membrane, the question of the scattering properties of the membrane arises. In previous works, we derived an approximation for the dispersion interaction for weakly responding materials by integrating over the volume of the dielectric object.^6^ Due to this volume integral, the interaction will strongly depend on the assumed thickness of the monolayer, which is not precisely quantifiable for monolayers. For h-BN, the thickness of a monolayer can be approximated by 0.3 nm.^46^ However, as we are interested in the behaviour at very short separations, the thickness's uncertainty will substantially impact the results.

To avoid issues concerning the monolayer's thickness, we separate the monolayer's surface into sections according to Fig. 2 with a rhomboidally shaped section (region I) surrounding the spherical hole with diameter *d*. According to the lattice structure, this section is defined by a side length *l* and a wedge angle *ϑ*. We used *ϑ* = 60° for h-BN. This section will be treated atomically, whereas the remaining outer part (yellow area; region II) is a non-contributing continuum by sufficiently increasing the size of the region I. This is motivated by the *r*^−6^ power law for the distance dependence of the short-range dispersive pairwise interaction potential. In region I, we consider an atomistic representation of the membrane characterised by the atom's positions ***r***_*i*_ and its type expressed by a local polarisability *α*_*i*_. This approach yields the discrete form of the first-order of the Born series expansion^21^9
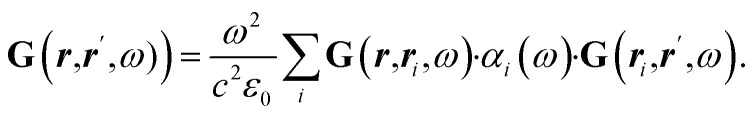


By plugging eqn (9) into the Casimir–Polder potential (8), the interaction in the region I can be written as the sum over the screened van der Waals interactions10
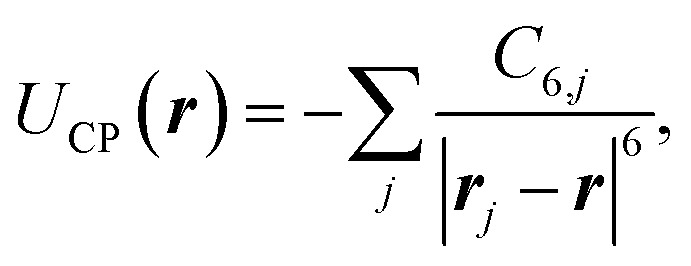
with the screened van der Waals coefficient from eqn (7).

Eqn (10) can be used as a criterion for the width of the region I. By considering a linear atomic chain with period *a*_C_ in a one-dimensional configuration, the total van der Waals potential for a particle at a distance *r* to the chain is determined by11
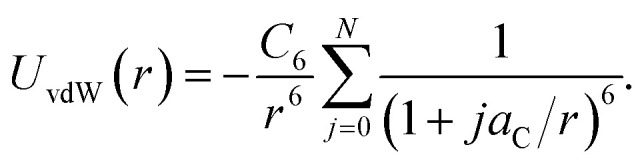


By restricting the chain to a finite particle number *N*, the deviation between the truncated and infinite sum can be obtained as12
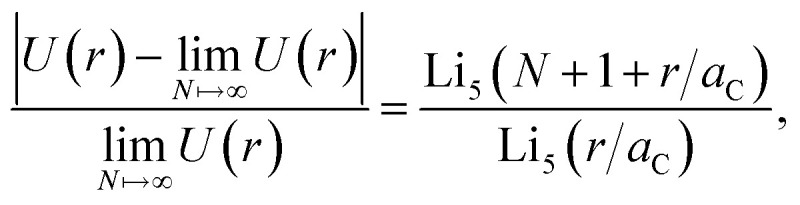
with the polylogarithm 
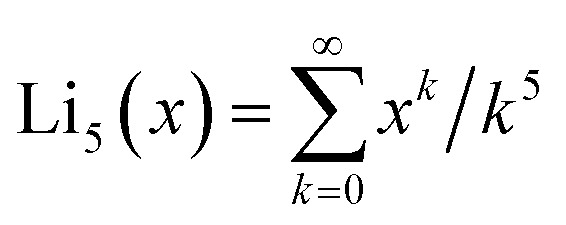
. Thus, the error according to the chain length *Na*_C_ can be estimated, leading to two atoms (*N* = 2) for an error below 1%. Consequently, two atomic rings surrounding the hole cause almost the entire interaction. For the holes considered here, all layers after the 3rd contribute to less than 1% of the total potential inside the holes.

A directional dependence appears assuming that ***α***_*i*_(*ω*) = *α*_*i*_(*ω*)***D***_*i*_,^47–49^ with ***D***_*i*_ being a 3 × 3-matrix, the Casimir–Polder potential can be written as13
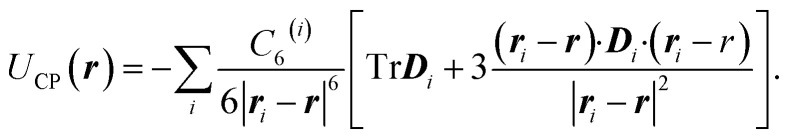


**Fig. 3:**
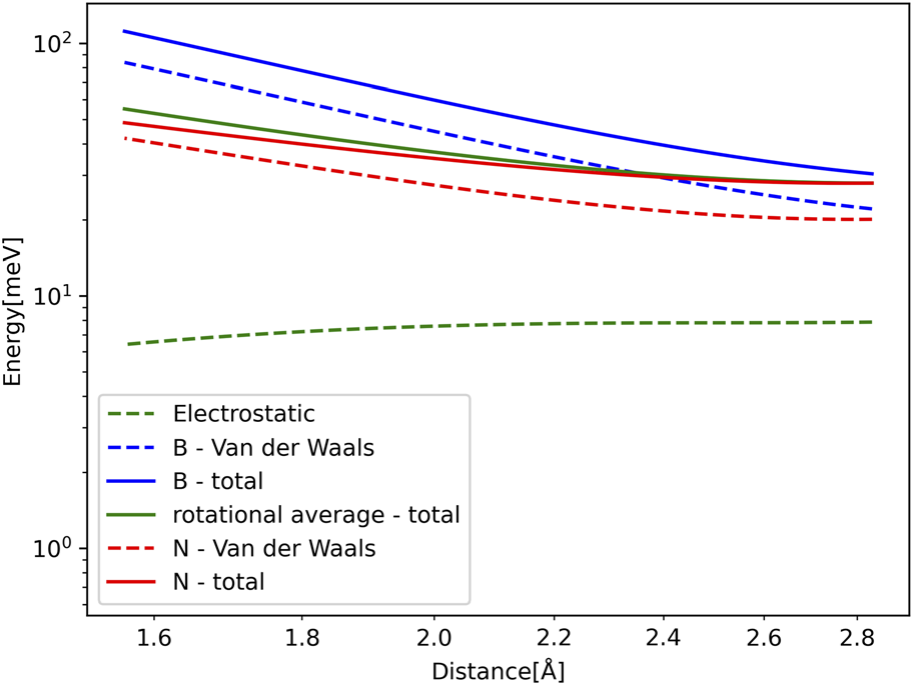
LogLog plot showing the potentials inside the 6 Å hole from Fig. 1. The lines show the potential from the holes' edges where boron and nitrogen atoms exist. It also shows the different contributions from electrostatic and van der Waals potentials.

These potentials inside hole (b) from Fig. 2 are shown in Fig. 3.

**Fig. 4:**
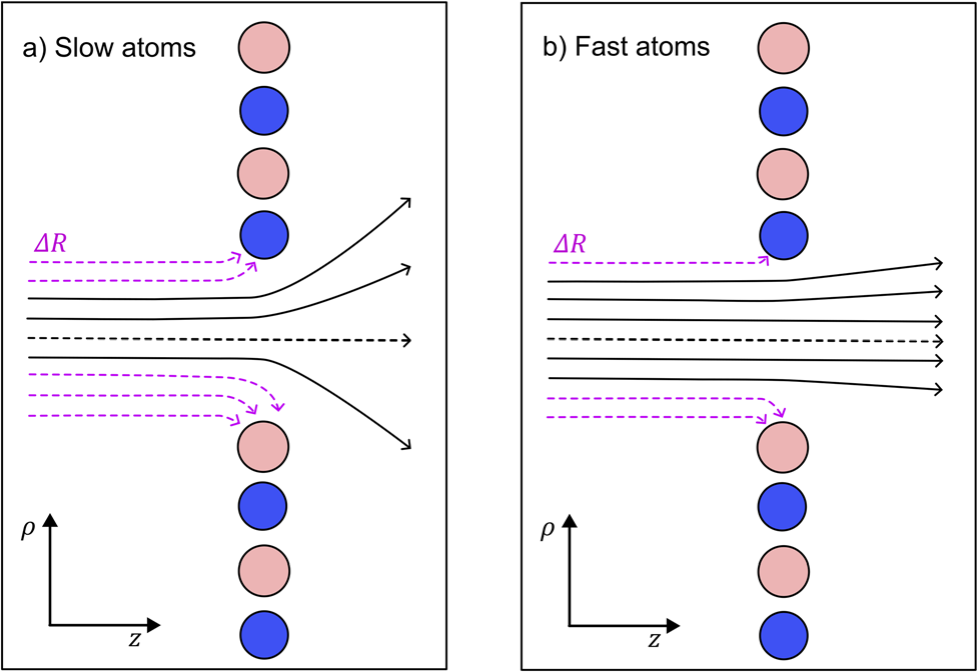
Schematic of the hole reduction Δ*R* for: (a) slow helium atoms, and (b) fast helium atoms passing through a hole in hexagonal boron nitride (h-BN). The dispersion forces are stronger for slow atoms compared to fast atoms. Therefore, the hole reduction effect is greater in part (a) compared to part (b). Additionally, the helium matter wave in part (a) experiences a greater angular spread compared to part (b). The transmission close to nitrogen atoms (blue) is increased compared to boron atoms (pink) since nitrogen atoms have a reduced C6 coefficient compared to boron atoms. This leads to interesting features in the phase shift plots and diffraction patterns.

### 2.5 Electrostatic forces

Such membranes are usually electrically neutral, like bulk systems. However, due to the removed atoms creating the hole, the single charges do not compensate each other near the hole. For this reason, each atom at the position ***r***_*i*_ also carries a charge *q*_*i*_, leading to an induced interaction^24,50^14
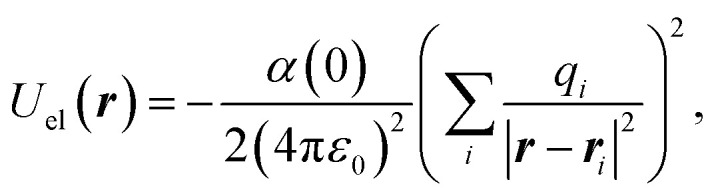
with the static polarisability of the free helium atom *α*(0). The charge *q*_*i*_ is obtained from Hirshfeld-partitioning^39^ of the electron density of h-BN. This leads to charges of +0.2|*e*| on B and −0.2|*e*| on N within pristine h-BN in good agreement with previous studies^51^ (there are severe disagreements for Bader^52,53^ charges in the literature^54–56^ as shown in ESI†). The atoms near to the hole show slightly increased local charges,^55^ never exceeding ±0.39|*e*|, however. The different potentials experienced by the helium atom inside the 6 Å hole from Fig. 1(b) are shown in Fig. 3. We can see that the van der Waals potential is considerably stronger. We will nevertheless also consider the electrostatic contributions in what follows.

## 3 Diffraction

We utilise Kirchhoff diffraction to find the resulting diffraction patterns. We need to find the hole reduction and phase shift caused by the van der Waals and electrostatic forces to do this. We find this by simulating a helium wavepacket passing a boron and a nitrogen atom and seeing how much of the wavepacket passes within the van der Waals radius. This then approximates the hole reduction caused by the surrounding atoms. Afterwards, we find the phase shift caused by the forces using an eikonal approximation as the atoms move very fast. Having both the phase shift and the hole reduction, we define a transmission function and find the diffraction patterns for the investigated holes.

### 3.1 Hole reduction

A known effect on atoms passing holes is a hole size reduction due to the forces on the atom attracting it to the edges.^6,57^ A previous method used to estimate the hole reduction is to track the classical trajectories of atoms and see how far away they have to pass from the wall to avoid collisions.^6^ Another method uses numerical simulations of the wavefront to find the diffraction at gratings.^57^ We will compare the classical trajectory method with numerically solving the propagation of the helium wavefunction colliding with an atom. We will simulate the collision with boron and nitrogen separately as it is very difficult to solve the propagation through the entire hole numerically. In addition, the forces become much weaker as you move away from the atom such that the vast majority of the force is caused by the closest atom in the monolayer, as demonstrated in eqn (11) and (12).

In both the classical and quantum mechanical approaches, we assume the extent of the atom to be equal to its van der Waals radius, which is 1.92 Å for B,^58^ and 1.55 Å for N.^59^ The h-BN layer is extended in *x*, *y* direction at *z* = 0. The classical hole reduction, Δ*R*_classical_, is then estimated by starting with the initial conditions ***r*** = (*x*,*y*,*z*), ***ṙ*** = (0,0,*v*), where *z* = 100 Å is the starting distance from the h-BN plane and *v* is the initial velocity of the He atom. Then we let ***r*** evolve according to

15 *m****r̈*** = −∇*U*(***r***),

with, *m* the mass of the helium. The potential

16 *U*(***r***) = *U*_el_(***r***) + *U*_vdW_(***r***)

consists of the electrostatic potential (14) reducing to17
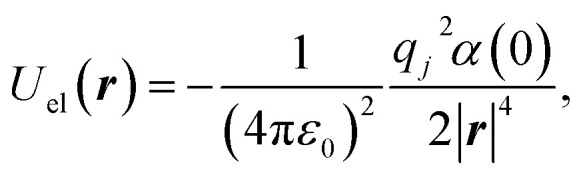
for a single atom *j*, with the static polarisability of the helium atom *α*(0) and the van der Waals potential from eqn (10) for a single atom *j*18
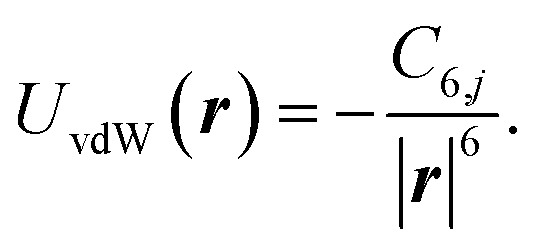


The atom propagates in the *z* direction, perpendicular to the monolayer. We test propagate with several different initial starting positions in the *x*, *y* coordinates. The hole reduction, Δ*R*_classical_, is then the smallest initial distance in the value of *x*, *y* plane where the atom does not pass within the van der Waals radius of the atom. The values of Δ*R*_classical_ are given for several velocities in Table 2.

**Table 2:** The hole reduction for helium passing by boron and nitrogen at different velocities. Both the classical results Δ*R*_classical_ and the quantum mechanical results Δ*R*_quantum_ are given

Atom	He velocity [ms^−1^]	Δ*R*_classical_ [Å]	Δ*R*_quantum_ [Å]
B	200	6.2	8.1
B	2000	2.5	3.6
B	20 000	1.9	2.3
N	200	5.9	7.8
N	2000	2.4	3.2
N	20 000	1.6	1.9

We also estimate the quantum mechanical hole reduction, Δ*R*_quantum_, by considering the case of a helium wave packet colliding with boron or nitrogen atoms. The wave packet, *ψ*, evolves according to the Schrödinger equation19
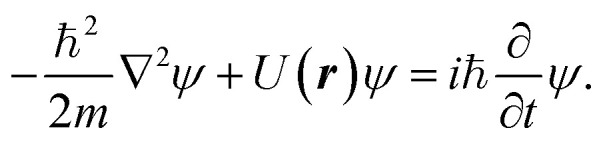


**Fig. 5:**
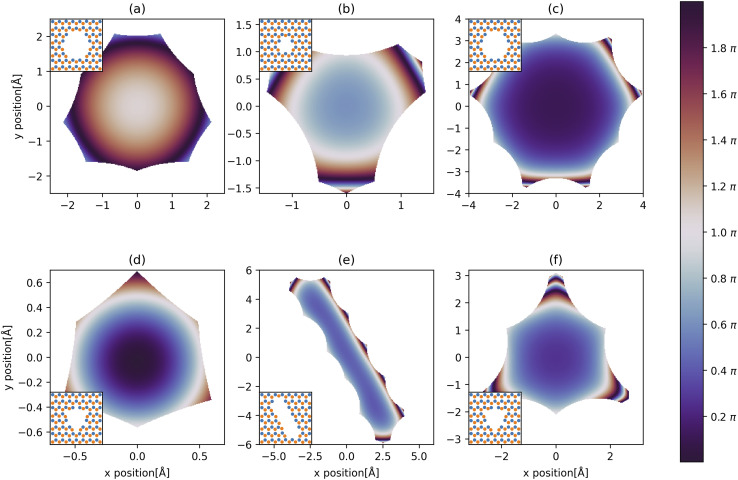
The phase shifts of the helium atoms from interactions with the atoms around the holes shown in Fig. 1. Black areas mean that the helium atom is not transmitted through the hole. The velocity of the matter wave in parts (a) and (d) is 2000 ms^−1^, whereas the velocity in parts (b), (c), (e) and (f) is 20 000 ms^−1^. The insets show the layout of the atoms around the hole with B in orange and N in blue. Due to the matter waves interaction with the monolayer, there is an additional reduction in transmission area of (a) 37%, (b) 31%, (c) 17%, (d) 88% (e) 5% and (f) 15% compared to the area of the monolayer covered by the removed atoms.

We used a finite difference scheme to evolve a wave packet by colliding with the atoms. The details of this scheme are detailed in the ESI.†^6,60–62^ Starting with a Gaussian wave packet of widths, *σ*_*r*_ = *σ*_*z*_ = 8 Å, representing a fifth of the box radius such that negligible amounts of the wave function are at the boundary of the simulation box. We move the potential at a given velocity *v* towards it from a distance of 60 Å to a distance of 40 Å past the helium wave packet. Finally, we assume that the parts of the wave packet that come within the van der Waals radius of the atom have collided with it. These parts of the wave packet might scatter, lose energy or otherwise lose coherence. We model this loss of coherence as an absorption of this section of the wave packet. The radius of the hole reduction then corresponds to the radius of the sphere that would absorb the same amount without any van der Waals or electrostatic interactions. We determine the hole reduction using the norm of the wave packet20
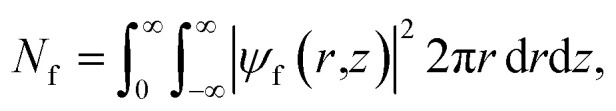
at the end of the propagation. We assume that the hole reduction corresponds to the radius of a moving sphere absorbing the part of the wave packet that comes within it such that21
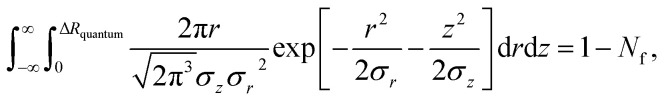
with *σ*_*r*_ and *σ*_*z*_ being the *r* and *z* spread of the wave packet. Solving eqn (21) for Δ*R*_quantum_ then gives22
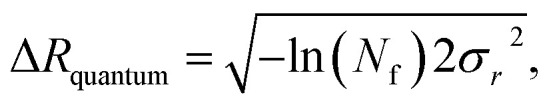
with the resulting hole reductions given in Table 2. In all cases, the quantum approach gives a larger hole reduction radius than the classical trajectory approach. The difference varies between a 19–44% increase in the reduction radius. For a small velocity of 200 ms^−1^, the hole reduction is so large that the holes in Fig. 1 are completely closed for the helium atom as the hole reduction is greater than the radius of the hole. Even for velocities of 2000 ms^−1^, the elliptic hole and the 6 Å hole, 1 (b) and (d) in Fig. 1, are too narrow for transmission of helium atoms. Only at velocities of 20 000 ms^−1^, all holes allow for some transmission. The resulting effective shape of the holes can be seen in Fig. 5.

### 3.2 Phase shift

By bypassing a dielectric obstacle, a matter wave experiences a spatial-dependent phase shift due to the interactions between both objects.^5,6,63,64^ This phase-shift reads23
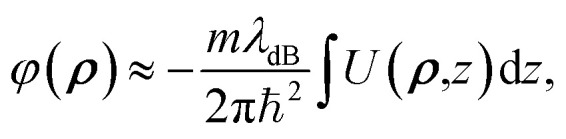
in eikonal approximation, where ***ρ*** = (*x*,*y*)^*T*^ are the in-plane coordinates, and *U*(***ρ***,*z*) is the potential experienced by the helium atom. *z* denotes the direction of the moving particles, and *λ*_dB_ = *h*/*mv* is the de Broglie wavelength. This approach means that the particles almost pass the obstacle in straight lines. Thus, the phase can be separated into three contributions: an electrostatic part24
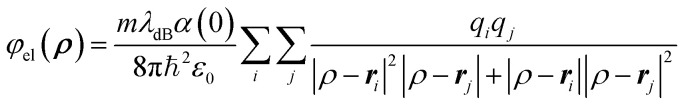
and van der Waals part25
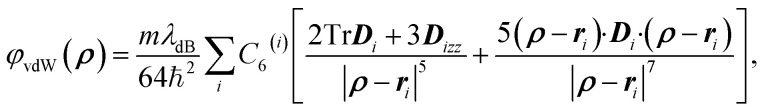
for the interaction with the atomic representation in region I.

We solve these equations to find the phase shift of a matter wave propagating through different types of holes in h-BN. The resulting phase shifts are plotted in Fig. 5, using a cyclical colour map. In parts (b), (c), (e) and (f), the He atoms have a high velocity (*v* = 20 000 ms^−1^), whereas in parts (a) and (d), the He atoms have a comparatively low velocity (*v* = 2000 ms^−1^). The de Broglie wavelength, *λ*_dB_, is inversely proportional to the velocity. Therefore, from eqn (23), we expect the phase shift to be smaller when the velocity of He atoms is higher (shorter *λ*_dB_).

This effect can be seen in Fig. 5, where the blue regions at the centre of the holes in parts (b), (c), (e) and (f) correspond to a low phase shift. The phase shift through the central region in part (b) is greater than in part (c) due to the smaller size of the hole (6 Å *vs.* 11 Å). Therefore, the matter wave experiences stronger dispersion interactions with the edge atoms. By comparison, there is a much larger phase shift at the centre of the holes in parts (a) and (d) due to the slower velocity (longer *λ*_dB_) of the matter wave. We can directly compare parts (a)/(c) and (d)/(f) to see this effect since the potentials around these pairs of holes are the same.

We also observe the h-BN lattice structure's effect in all the phase shift plots. Boron atoms have a higher dispersion coefficient compared to nitrogen atoms. Therefore, we observe greater transmission and more fringes near nitrogen-terminated edges in all cases. For example, this can be clearly seen in the elliptical hole in part (e), where the nitrogen-terminated edge (long right edge) has features that are not observed on the boron-terminated edge (left long edge).

Another example is the snowflake structure in part (f), which has unequal transmission through the different arms of the snowflake. The structure has 6 arms, whereas only 3 arms strongly transmit matter waves. In each arm, there are either 3 nitrogen edge atoms or 3 boron edge atoms (these alternate for each arm around the hole). We observe a high transmission in the arms where nitrogen atoms are dominant, along with rapidly oscillating phase shift patterns. It could be interesting to compare this result with an atomically homogeneous structure, such as graphene, where we would expect to see a 6-fold, rather than 3-fold, rotational symmetry in the phase shift pattern.

By comparison, in part (d), we do not observe the rapidly oscillating features present in part (f). We attribute this to the lower velocity of the matter wave in part (d) compared to (f), which leads to stronger dispersion interactions and reduced transmission through the arms of the snowflake.

### 3.3 Diffraction patterns

From the phase shifts and the hole reduction, we can derive a transmission function *T*(***ρ***), which is e^*iφ*(***ρ***)^ wherever the helium atom is transmitted through the hole and zero everywhere else. The diffraction pattern from passing a hole is given by Kirchhoff's diffraction formula:^65^26
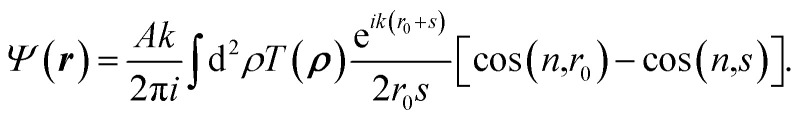
Here, *A* is the amplitude of the wave function, and *k* = 2π/*λ*_dB_. *r*_0_ is the distance between the point source of the helium matter wave and the in-plane coordinates ***ρ*** of the h-BN monolayer. *s* is the distance between ***ρ*** and the coordinates ***r*** at which the diffraction pattern is measured (*s* = |***r*** − ***ρ***|). The sum of *r*_0_ and *s* describes the total path length of the helium matter-wave. cos(*n*,*r*_0_) and cos(*n*,*s*) describe the cosine similarity between *r*_0_, *s* and the normal *n* of the h-BN plane. The cosine similarity is defined as27
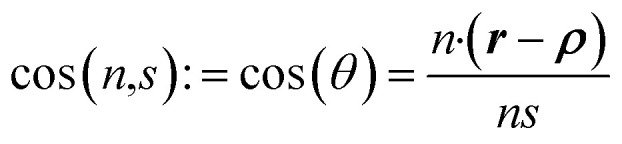
with an equivalent expression for cos(*n*,*r*_0_).

If the path length of the matter wave is large compared to the linear dimensions of the aperture, we can apply the small angle approximation, whereby [cos(*n*,*r*_0_) − cos(*n*,*s*)] → 2 cos(*δ*). Here, *δ* is the angle between the helium matter wave's displacement vector and the h-BN plane's normal vector. We can further assume that the distances *r*_0_ and *s* are measured from the origin of the coordinate system rather than an arbitrary point in the h-BN plane. These transformed distances are 
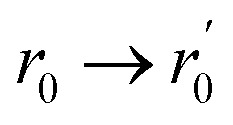
 and *s* → |***r***|. This leads to the Fraunhofer approximation,^65^28
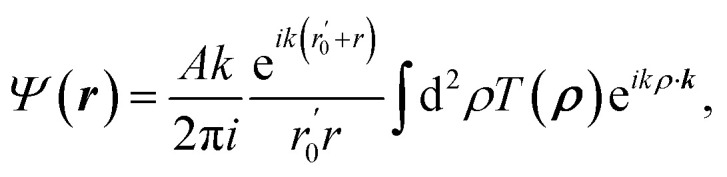
where we have assumed that cos(*δ*) ≈ 1 for normal incidence. The diffraction pattern at a screen is then given by the absolute value squared of *ψ*(***r***)29
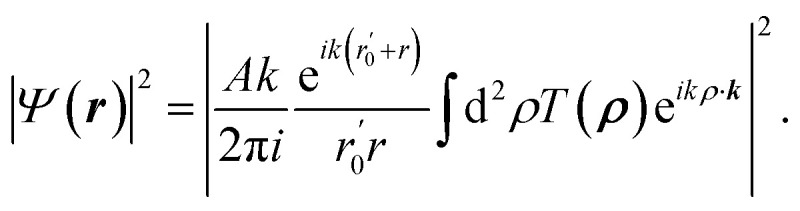


**Fig. 6:**
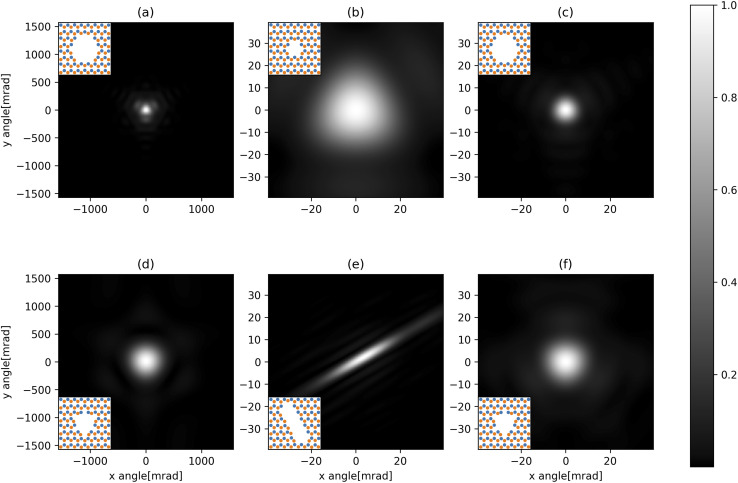
Diffraction pattern resulting from a helium atom passing the holes in Fig. 1 normalised to the maximum intensity. The patterns are 1 m away from the holes. (a) and (d) have a velocity of 2000 ms^−1^, and (b), (c), (e) and (f) have a velocity of 20 000 ms^−1^. The insets show the layout of the atoms around the hole with B in orange and N in blue.

The resulting diffraction patterns, normalised such that the maximum value is one, are shown in Fig. 6. When the velocity of the helium atom is 2000 ms^−1^, the angular spread is very large so it should be noted that the Fraunhofer approximation holds less well.

Fig. 6 shows that the h-BN lattice structure directly affects the diffraction patterns. In all cases, by comparing the patterns from Fig. 5, we see that there are more fringes close to nitrogen atoms, which have a lower dispersion coefficient compared to boron atoms. This is clearly visible in parts (a), (b), (c) and (f), where we observe a 3-fold, rather than 6-fold rotational symmetry in the phase-shift plot.

Part (d) has a much more uniform phase shift across the hole; thus, the diffraction pattern is more similar to how light would diffract through a similarly shaped hole. In part (f), which is the same hole only diffracted by faster atoms, we see a much clearer fringe pattern as we also have diffraction through some of the arms of the snowflake where there are atoms on almost all sides.

For narrow slits, such as in part (e), we would expect the diffraction pattern to spread more along the short axis of the hole compared to the long axis. While we see this, we can also see that the diffraction pattern is stretched along the right side of the short axis.

**Fig. 7:**
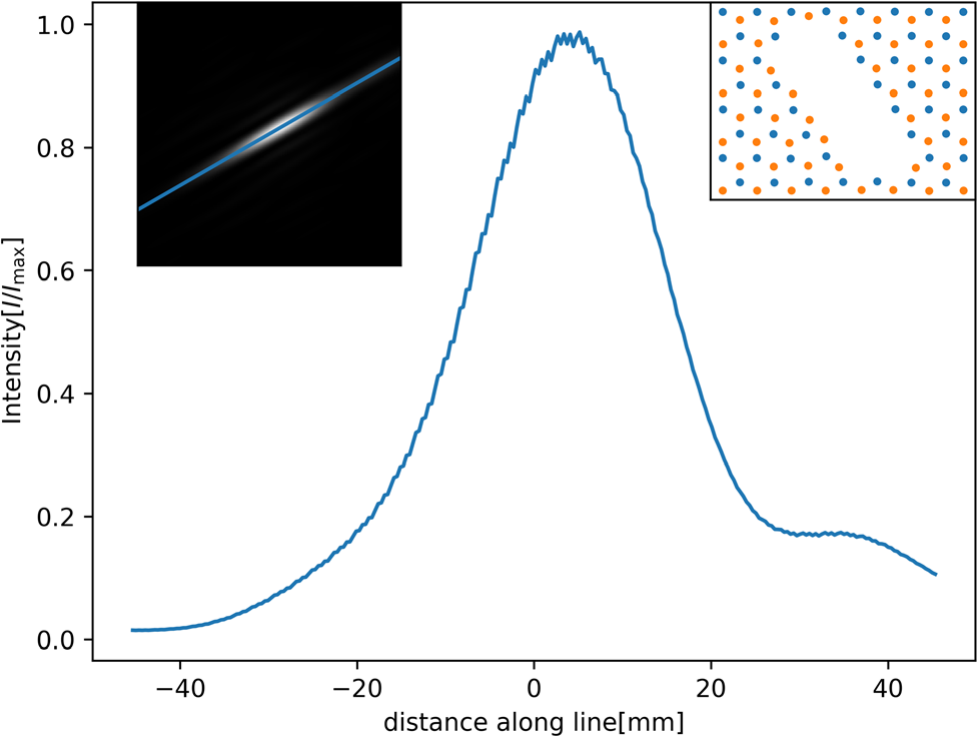
Diffraction pattern along the indicated line for the elliptic hole (Fig. 6(e)). We clearly see that more atoms go to the right side compared to the left. The centre of the pattern is also shifted right. The insets show the line we plot along and the layout of the atoms around the hole, with B in orange and N in blue.

Even though the nitrogen atoms have a larger polarizability enhancement, they still have a smaller dispersion coefficient compared to boron atoms. Therefore, there is some asymmetry in the transmission function, leading to a diffraction pattern that is not centred directly in the middle of the elliptical hole but that is shifted closer to the nitrogen-terminated edge. Essentially, the helium atoms are gaining angular momentum from one side, having a stronger potential and being “shot” right. This is illustrated in Fig. 7.

## 4 Conclusions

This paper describes a method of determining diffraction patterns from matter waves passing through holes in monolayer materials, using h-BN as an example. Our DFT calculations reveal that the removal of atoms to create the hole produces a “polarisability ripple” around the defect where the atoms surrounding the defect show enhanced polarisability, whereas the atoms in the next ring show reduced polarisability. This oscillatory behaviour diminishes rapidly with increasing distance to the defect.

Based on these polarisabilities, we have estimated the hole reduction and phase shift resulting from van der Waals and electrostatic interactions between the atom and the h-BN monolayer through a numerical wave packet propagation considering the edge atom nearest to the classical trajectory of the helium scattering path. We find the van der Waals contribution to dominate the scattering potential as compared to the electrostatic part. Using macroscopic diffraction theory, the propagation allowed us to find diffraction patterns of holes smaller than 1 nm in h-BN. We found that the predicted atomic polarizabilities and dispersion coefficients have a significant effect on the diffraction patterns, such as shifting the elliptical holes' diffraction pattern in the direction of the nitrogen-terminated edge. In terms of applications to binary holography, we have shown that is possible to diffract at sub-nanometer holes. Our results therefore suggest that matter-wave lithography could potentially achieve sub-nanometer resolution by using 2D monolayer materials as a mask. The next step will be to test the model in real experimental studies. This will require preparation of the required hole shapes, which is challenging with current technology. A viable method could be electron radiation using aberration-corrected transmission electron microscopy (ACTEM), which has recently been used to demonstrate the creation of single vacancies in h-BN.^66^ Other possible paths include helium ion milling.^27^ In order to avoid issues with material defects as much as possible, it is important that experiments are carried out using large area single crystal material.^67^

## Author contributions

E. Z. performed the DFT calculations. E. K. O. calculated dispersion forces and diffraction optics. E. K. O. and E. Z. analysed the results. J. F. conceptualised the project. J. F., B. H. and M. W. acquired funding for and supervised the project. All authors contributed to writing the manuscript.

## Conflicts of interest

There are no conflicts to declare.

## Supplementary Material

NA-006-D4NA00322E-s001

## Data Availability

The data supporting this article is all included in the manuscript and the ESI.†
