# Supplementary material for: Atomic diffraction by nanoholes in hexagonal boron nitride
Source: Nanoscale Adv. 2024 Sep 2;6(21):5337–47. doi: 10.1039/d4na00322e (PMC11367323; doi:10.1039/d4na00322e)
Supplement: NA-006-D4NA00322E-s001 [file NA-006-D4NA00322E-s001.pdf]

Supplementary Information (SI) for Nanoscale Advances.
This journal is © The Royal Society of Chemistry 2024

Atomic diffraction by nanoholes in hexagonal boron nitride Supplementary information

Eivind Kristen Osestad,[a,b] Ekaterina Zossimova,[c,d] Michael Walter,[d,e] Bodil Holst,[a,b] and Johannes Fiedler[a]

April 12, 2024

Contents

S1 Wave propagation simulation **1**

S2 Atomic charges in hBN **3**

S1 Wave propagation simulation

We want to estimate how much the effective size of our holes shrink due to the dispersion forces as this is a know effect in other holes[1]. We used two methods to do this. Here we give a more detailed description of the second method which is a finite difference Cranck-Nicholson scheme.

To estimate the reduced radius of the holes we will simulate a helium wave-packet colliding with the boron and nitrogen atoms. We will assume that any part of the wave packet that comes within the Van der Waals radius of the boron and nitrogen is absorbed and disappears. Outside the VdW radius, we assume that the atom obeys the Schrödinger equation with a Van der Waals potential and an electrostatic potential

$$\frac{\hbar^2}{2m}\nabla^2\psi(\boldsymbol{r},t) - \frac{C_6}{|\boldsymbol{r}-\boldsymbol{r}_A|^6}\psi(\boldsymbol{r},t) - \frac{1}{4\pi\varepsilon_0}\frac{q^2\alpha(0)}{|\boldsymbol{r}-\boldsymbol{r}_A|^4}\psi(\boldsymbol{r},t) = i\hbar\frac{\partial}{\partial t}\psi(\boldsymbol{r},t)\,, \tag{S1}$$

with $\boldsymbol{r}_A$ being the position of the nitrogen or boron atom, $\boldsymbol{r}$ being the position in the simualted box,

The general form of the Schrödinger equation is

$$\hat{H}\psi = i\hbar\frac{\partial}{\partial t}\psi\,, \tag{S2}$$

which has a solution on the form

$$\psi(\boldsymbol{r},t) = \exp(-\frac{i\hat{H}}{\hbar}(t-t_0))\psi(\boldsymbol{r},t_0)\,. \tag{S3}$$

A half implicit half explicit finite difference method is then used to approximate the propagation

$$(1+\frac{i\hat{H}}{2\hbar}\delta t)\psi_{t+\delta t} = (1-\frac{i\hat{H}}{2\hbar}\delta t)\psi_t\,. \tag{S4}$$

[0a] *Department of Physics and Technology, University of Bergen, Allégaten 55, 5007 Bergen, Norway.; E-mail: johannes.fiedler@uib.no*
[0b] *Lace Lithography AS, Allégaten 55, 5006 Bergen, Norway.*
[0c] *Department of Physics and Astronomy, Living Systems Institute, University of Exeter, EX4 4QD, Exeter, UK.*
[0d] *Freiburg Center for Interactive Materials and Bioinspired Technologies (FIT), University of Freiburg, D-79110 Freiburg, Germany.*
[0e] *Institute of Physics, University of Freiburg, Hermann-Herder-Str. 3, 79104 Freiburg, Germany.*

This finite difference scheme is the same as used by [2], we use it as it is norm preserving. Now we define

$$(1 \pm \frac{i\hat{H}}{2\hbar}\delta t) = \mathcal{A}_{\pm}\,, \tag{S5}$$

such that equation S4 becomes

$$\mathcal{A}_{+}\psi_{t+\delta t} = \mathcal{A}_{-}\psi_{t}\,. \tag{S6}$$

To reduce the number of points having to be simulated we assume the helium atom is stationary and that the potential is moving towards it. In addition, we assume that the collision is head-on and that there is no angular momentum relative to the helium atom. Thus we can model a box around the helium atom at rest and move the boron or nitrogen into the box during the simulation. We can also assume that the wavefunction can be split into an angular part which we assume is uniform and a radial and z-component part which evolves in time. The resulting Schrödinger equation then looks like

$$\frac{\hbar^2}{2m}(\frac{\partial^2}{\partial z^2} + \frac{\partial^2}{\partial r^2} + \frac{1}{r}\frac{\partial}{\partial r})\psi - \frac{C_6}{|\boldsymbol{r} - \boldsymbol{r}_A|^6}\psi - \frac{1}{4\pi\varepsilon_0}\frac{q^2\alpha(0)}{|\boldsymbol{r} - \boldsymbol{r}_A|^4}\psi = i\hbar\frac{\partial}{\partial t}\psi\,. \tag{S7}$$

We define

$$V(\boldsymbol{r}) = -\frac{C_6}{|\boldsymbol{r}|^6} - \frac{1}{4\pi\varepsilon_0}\frac{q^2\alpha(0)}{|\boldsymbol{r}|^4}\,, \tag{S8}$$

thus equation S7 becomes

$$\frac{\hbar^2}{2m}(\frac{\partial^2}{\partial z^2} + \frac{\partial^2}{\partial r^2} + \frac{1}{r}\frac{\partial}{\partial r})\psi + V(\boldsymbol{r} - \boldsymbol{r}_A)\psi = i\hbar\frac{\partial}{\partial t}\psi\,. \tag{S9}$$

We divide the box around the helium atom into a uniform grid. We organize the wavefunction into a $n \cdot m$ vector organized such that

$$\psi_1 = \psi(r_1, z_1)\,,\ \psi_2 = \psi(r_2, z_1)\,,\ \ldots\,, \tag{S10}$$

$$\psi_n = \psi(r_n, z_1)\,,\ \psi_{n+1} = \psi(r_1, z_2)\,,\ \ldots\,, \tag{S11}$$

$$\psi_{n\cdot m-1} = \psi(r_{n-1}, z_m)\,,\ \psi_{n\cdot m} = \psi(r_n, z_m)\,. \tag{S12}$$

In our case we use n = 256 and m = 256. With r going from 0nm to 8nm and z going from -4nm to 4nm, that makes $\delta r = 7.8$pm and we use a timestep of $\frac{dr}{32 v_z}$ with v_z being the velocity of the helium atom.

The operator $\mathcal{A}_{+} = (1 + \frac{i\hat{H}}{2\hbar}\delta t)$ can then be expressed as a $m \cdot n \times m \cdot n$-matrix. The resulting matrix is a block tridiagonal matrix

$$\mathcal{A}_{+} = \begin{bmatrix} D_1 & U_1 & 0 & 0 & \dots & 0 & 0 & 0 \\ L_2 & D_2 & U_2 & 0 & \dots & 0 & 0 & 0 \\ 0 & L_3 & D_3 & U_3 & \dots & 0 & 0 & 0 \\ \vdots & \vdots & \vdots & \vdots & \ddots & \vdots & \vdots & \vdots \\ 0 & 0 & 0 & 0 & \dots & L_{m-1} & D_{m-1} & U_{m-1} \\ 0 & 0 & 0 & 0 & \dots & 0 & L_m & D_m \end{bmatrix}, \tag{S13}$$

with

$$U_i = L_i = -i\frac{\hbar\delta t}{4m\delta z^2}\mathbf{1}_{n\times n}\,, \tag{S14}$$

and

$$D_i = \begin{bmatrix} d_{i,1} & u_1 & 0 & 0 & \dots & 0 & 0 & 0 \\ l_2 & d_{i,2} & u_2 & 0 & \dots & 0 & 0 & 0 \\ 0 & l_3 & d_{i,3} & u_3 & \dots & 0 & 0 & 0 \\ \vdots & \vdots & \vdots & \vdots & \ddots & \vdots & \vdots & \vdots \\ 0 & 0 & 0 & 0 & \dots & l_{n-1} & d_{i,n-1} & u_{n-1} \\ 0 & 0 & 0 & 0 & \dots & 0 & l_n & d_{i,n} \end{bmatrix}, \tag{S15}$$

where

$$d_{i,j} = 1 + (\frac{\hbar^2}{m\delta r^2} + \frac{\hbar^2}{m\delta z^2} + V_{i,j})\frac{i\delta t}{2\hbar}\,, \tag{S16}$$

$$u_j = -i\frac{\hbar\delta t}{4m\delta r^2} - i\frac{1}{j}\frac{\hbar\delta t}{2m\delta r^2}\,, \tag{S17}$$

$$l_j = -i\frac{\hbar\delta t}{4m\delta r^2} + i\frac{1}{j}\frac{\hbar\delta t}{2m\delta r^2}\,, \tag{S18}$$

$$u_1 = 0\,. \tag{S19}$$

The operator $\mathcal{A}_-$ is the complex conjugate of $\mathcal{A}_+$.

Performing a time step is therefore equivalent to solving the matrix equation

$$\mathcal{A}_+\boldsymbol{\psi}_{t+\delta t} = \boldsymbol{b}_t, \tag{S20}$$

with

$$\boldsymbol{b}_t = \mathcal{A}_-\boldsymbol{\psi}_t\,. \tag{S21}$$

can then be solved by iteration if certain criteria are met[3]. The block matrix is solved by iteratively by solving

$$D_i\boldsymbol{x}_i = \boldsymbol{b} - L_i\boldsymbol{x}_{i-1} - U_i\boldsymbol{x}_{i+1}\,, \tag{S22}$$

which should have $\boldsymbol{x}$ converge towards a solution[3]. Since D_i is a tridiagonal matrix it can be solved quickly using the tridiagonal matrix algorithm [4].

At the end of each time step we multiply the parts inside the van der Waals radius of the atom by 0, that is $|\boldsymbol{r} - \boldsymbol{r}_A| < r_{\mathrm{vdW}}$, and we move the box according to according to its velocity, $\boldsymbol{r}_{A,new} = \boldsymbol{r}_A - \boldsymbol{v}\delta t$, with $\boldsymbol{v}$ being the velocity of the helium atom.

We do a total of 6 runs, 3 different velocities for nitrogen and 3 different velocities for boron. The starting shape of the wavepacket is such that the wavepacket sqared is a Gaussian

$$\psi_0(\boldsymbol{r}) = \frac{1}{\sqrt{\sqrt{2\pi^3}\sigma_z\sigma_r^2}}\exp\left[-\frac{r^2}{4\sigma_r} - \frac{z^2}{4\sigma_z}\right]\,, \tag{S23}$$

with $\sigma_r = \sigma_z = 8$Å. We start the propagation with the center of our box at 60Å before the N or B atom and end it at 40Å past the atoms. The final wavefunction is then compared to the first one to find the amount that passed within the van der Waals radius.

S2 Atomic charges in hBN

The definition of atomic charges in molecules suffers from a degree of arbitrariness, such that different schemes for the determination have been devised. Tab. 1 compares values obtained from Bader and Hirshfeld partitioning from the literature and our calculations. All results show the same qualitative trend in that the nitrogen atom is negatively charged and the boron atom is positively charged contrary to chemical understanding. Generally the charges determined by the Bader method are larger than these from Hirshfeld partitioning.

There is a large deviation between Bader results determined from different DFT codes, but the Hirshfeld values are in much better agreement. The origin of the difference in the Bader values is beyond the scope of this investigation. We therefore use the Hirshfeld charges in our manuscript due to their consistency. Atomic charges near to the holes appear to be larger than these of pristine hBN. The maximal Hirshfeld charges we find do not exceed $\pm 0.39|e|$, however.

Table 1: Charges (in terms of unit charge $|e|$) around atoms in h-BN.

**Material**	**N Charge**	**B Charge**	**Method**
hBN pristine[5]	-0.47	+0.47	Bader VASP
hBN pristine[6]	-0.67	+0.67	Bader VASP
hBN pristine[7]	-2.28	+2.28	Bader GPAW
hBN pristine[8]	-0.17	+0.17	Hirshfeld Abinit
hBN pristine ours	-2.19	+2.19	Bader
hBN pristine ours	-0.20	+0.20	Hirshfeld
PQP+[9]	-1.1		Bader GPAW
N edge in hBN hole[6]	-1.59		Bader VASP
B edge in hBN hole[6]		+1.50	Bader VASP
Circular hole (6 Ang) ours	-0.39	0.38	Hirshfeld
Circular hole (10 Ang) ours	-0.32	0.32	Hirshfeld
Elliptical hole ours	-0.39	0.38	Hirshfeld
Snowflake hole ours	-0.29	0.24	Hirshfeld

References

[1] Johannes Fiedler and Bodil Holst. An atom passing through a hole in a dielectric membrane: impact of dispersion forces on mask-based matter-wave lithography. *Journal of Physics B: Atomic, Molecular and Optical Physics*, 55(2):025401, jan 2022.

[2] Emanuele Galiffi. Quantum reflection in two dimensions, 2015.

[3] W Yang, K Li, and K Li. A parallel solving method for block-tridiagonal equations on cpu–gpu heterogeneous computing systems. *The journal of supercomputing*, 73:1760–1781, 2017.

[4] P. Niyogi. *Introduction to Computational Fluid Dynamics*. Pearson Education Canada, 2006.

[5] Rui Wang, Jiali Yang, Xiaozhi Wu, and Shaofeng Wang. Local charge states in hexagonal boron nitride with Stone-Wales defects. *Nanoscale*, 8(15):8210–8219, April 2016.

[6] Li-Chang Yin, Hui-Ming Cheng, and Riichiro Saito. Triangle defect states of hexagonal boron nitride atomic layer: Density functional theory calculations. *Phys. Rev. B Condens. Matter Mater. Phys.*, 81(15):153407, April 2010.

[7] Hajime Suzuki, Itsuki Miyazato, Tanveer Hussain, Fatih Ersan, Satoshi Maeda, and Keisuke Takahashi. Designing two-dimensional dodecagonal boron nitride. *CrystEngComm*, 24(3):471–474, 2022.

[8] B Altintas, C Parlak, C Bozkurt, and R Eryiğit. Intercalation of graphite and hexagonal boron nitride by lithium. *Eur. Phys. J. B*, 79(3):301–312, February 2011.

[9] Kang Cui, Kunal S. Mali, Oleksandr Ivasenko, Dongqing Wu, Xinliang Feng, Michael Walter, Klaus Müllen, Steven De Feyter, and Stijn F. L. Mertens. Squeezing, then stacking: From breathing pores to three-dimensional ionic self-assembly under electrochemical control. *Angew. Chem. Int. Ed.*, 53(47):12951–12954.
